# Clinical Relevance of Nuclear Magnetic Resonance LipoProfile

**DOI:** 10.3389/fnume.2022.960522

**Published:** 2022-07-13

**Authors:** Emmanuel U. Emeasoba, Emeka Ibeson, Ifeanyi Nwosu, Nadine Montemarano, Jacob Shani, Vijay S. Shetty

**Affiliations:** ^1^Department of Medicine, Maimonides Medical Center, New York, NY, United States; ^2^Heart and Vascular Institute, Maimonides Medical Center, New York, NY, United States

**Keywords:** cardiovascular risk, cholesterol, nuclear magnetic resonance, lipoprotein particles, lipid panel

## Abstract

Identifying risk factors for cardiovascular diseases in patients is key to reducing their resulting morbidity and mortality. Currently, risk factors are assessed using parameters that include and emphasize the role of the level of cholesterol carried by lipoproteins. Most providers focus on targeting cholesterol levels in patient management. However, recent research shows that lipoprotein particle number is more predictive of cardiovascular risk than cholesterol levels. The Nuclear Magnetic Resonance (NMR) LipoProfile test assesses the number of lipoprotein particles, sizes of lipoproteins, levels of cholesterol, and patient risk categories. Furthermore, it enables the identification of patients with underestimated cardiovascular risks—those with a discordant high number of low-density lipoprotein (LDL) particles (LDL-P) despite low cholesterol levels. While the NMR LipoProfile test requires a higher cost and longer waiting time for results in comparison to the lipid panel test, its advantages cannot be ignored. This review article focuses on exploring the routine use of NMR LipoProfile in clinical practice.

## Highlights

- Lipoprotein particle number predicts cardiovascular risk better than cholesterol.
- Nuclear magnetic resonance LipoProfile assesses lipoprotein particle number.
- Using nuclear magnetic resonance LipoProfile clinically could improve patient care.

## Introduction

NMR LipoProfile is a fairly new and more accurate method of assessing cardiovascular risk in individuals than the more widely used lipid panel test. It differs from the lipid panel in that it measures other parameters that are indicative of cardiovascular risk status such as lipoprotein particle numbers. This paper aims to describe the NMR LipoProfile and discuss its relevance in cardiovascular settings.

### Background on Lipoprotein

Cardiovascular diseases (CVD) are the leading causes of death in the United States, attributable to 25% of deaths each year (1–3). Identifying cardiovascular risk factors and deciding on the best treatment modalities to manage or mitigate these risks is usually the goal of the physician in a bid to reduce mortality and promote quality of life. Lipoprotein particles which include LDL, high-density lipoproteins (HDL), and very-low-density lipoprotein (VLDL) are the transport protein molecules for cholesterol in the peripheral system. These lipoprotein particles are made up of combinations of protein, cholesterol, triglyceride, and phospholipid molecules and have been established as key clinical indices that aid in both assessment and management of CVD risk. Characteristics of lipoprotein that contribute to increased CVD risk include abnormal levels of cholesterol being transported, the distribution of specific lipoprotein subclass, as well as abnormal distributions of the lipoprotein particles themselves even when cholesterol levels are normal (4, 5).

LDL-P could either be small or large and situations, where small LDL-P are abundant, may be associated with increased atherogenic risk than situations where large LDL particles are plentiful (6–8). Small LDL-P occur as a result of metabolic action on lipoproteins rich in triglycerides such as VLDL. They are also predominant in individuals with hyperbetalipoproteinemia and those with insulin resistance including patients diagnosed with type II diabetes mellitus, hypertriglyceridemia, obesity, and hypertension. Small dense LDL is thought to be more atherogenic because they are better able to penetrate the endothelial cell barrier and enter the intima. They are more susceptible to oxidation, bind to proteoglycans in the arterial wall, and have a longer half-time in the circulation than large LDL-P. Other distributions of lipoprotein subclasses that confer an increased cardiovascular risk include low levels of HDL particles and high levels of VLDL. In CVD assessment, lipoproteins are used in addition to other indicators of cardiovascular risk such as gender, smoking status, and diabetes (6, 9). During risk management, treatment goals and success hinge on pre-set lipoprotein levels, and in this case, clinical decision-making is almost solely dependent on measured lipoprotein levels (6, 10).

Although both lipoprotein cholesterol and lipoprotein particle levels have been known to confer risk, LDL-P number/concentration has been identified in multiple studies as the strongest predictor of future cardiovascular events when compared to LDL cholesterol (LDL-C) and apolipoprotein B (6, 11–15). Data analysis from the Framingham study showed an association between LDL particle number and increased cardiovascular risk in both men (HR = 1.24: 1.10–1.39) and women (HR = 1.33: 1.17–1.50). While the association between LDL-C and increased cardiovascular risk was only seen in women (HR = 1.18: 1.02–1.37) and not in men (HR = 1.06: 0.94–1.20) (6). Apolipoprotein B levels and LDL-P numbers are more strongly associated with atherosclerotic cardiovascular risk score (ASCVD) than LDL-C, particularly when the levels of LDL-C and Apolipoprotein B levels or LDL-P numbers are discordant. Guidelines for treatment include recommendations for specific LDL-C levels as the goal of treatment, however, LDL-P numbers may be a better indicator of risk than LDL-C. Focusing on the LDL-P number as the treatment target may be more helpful in providing individualized treatment modalities, as it could potentially distinguish patients whose risks have not been adequately managed from those with an adequate response to therapy (6).

A key subset of individuals who will likely benefit from lipoprotein particle measurements are those with discordance (having low LDL-C but high LDL-P) (6, 16). One study conducted with a community sample showed that although a large percentage of individuals (79%) with low LDL-C had corresponding low LDL-P numbers, about 21% had high LDL-P numbers (6). The rates may even be higher in clinical samples as evidenced by a finding of discordance in approximately 63% of patients in a lipid clinic (16). Factors associated with discordance include male gender and smoking status, lipid-lowering therapy, and patients approaching their target LDL-C. As the number of LDL lower with treatment, LDL particles get more cholesterol depleted leading to this discordance (17). Furthermore, those with this discordance had higher CVD risk including a lower probability of event-free survival and incident CVD compared to concordant individuals (6, 18). In a study where LDL-C and LDL-P were associated with incident CVD overall, among those with discordant levels, only LDL-P was significantly associated with incident CVD (HR = 1.45: 1.19–1.78) unlike LDL-C (HR = 1.07: 0.88–1.30) (18). Because patients with discordance tend to have higher CVD risk, they will likely benefit more from intensive therapy targeted toward lowering their LDL particle number than their LDL-C level (6, 18).

Besides risk stratification, assessing lipoprotein numbers can also identify beneficial states in patients. HDL particle number is cardioprotective for patients. Specifically, the larger HDL particles confer this cardiovascular benefit. Also, a low LDL-P number is a better indicator of low CVD risk than low LDL-C, thereby emphasizing its usefulness as a target of lipid management (6). Therefore, stratifying a patient's lipoprotein profile will help provide an individualized approach to managing patients with varying degrees of cardiovascular risk.

### Introduction to NMR LipoProfile Test and How It Is Different From the Standard of Care

As discussed earlier, the majority of lipoprotein measurements focus on measuring the cholesterol content of lipoproteins (6, 19). The most commonly used test for assessing lipoprotein-related cardiovascular risk measures the levels of cholesterol and triglycerides carried by LDL, HDL, and VLDL. This test involves utilizing enzymatic or photometric procedures to measure the amount of cholesterol that is carried by these lipoproteins which are thought to be an estimate of the number of the lipoprotein particles (20). While the amount of cholesterol carried by the lipoproteins can give a picture of an individual's cardiovascular status, measurement of the lipoprotein particle number is a more accurate measure of cardiovascular risk. Studies have shown that there is a wide variance in the cholesterol content of lipoproteins among individuals leading to differences between the measured cholesterol level and the lipoprotein particle number (6, 21). As such, even the most accurate measure of lipoprotein cholesterol amounts fails to accurately determine the number of circulating lipoproteins for these individuals. The main reason is the variations in chemical composition, density, and size of lipoproteins limit the characterization of lipoprotein content by conventional blood tests (22).

**Figure 1:**
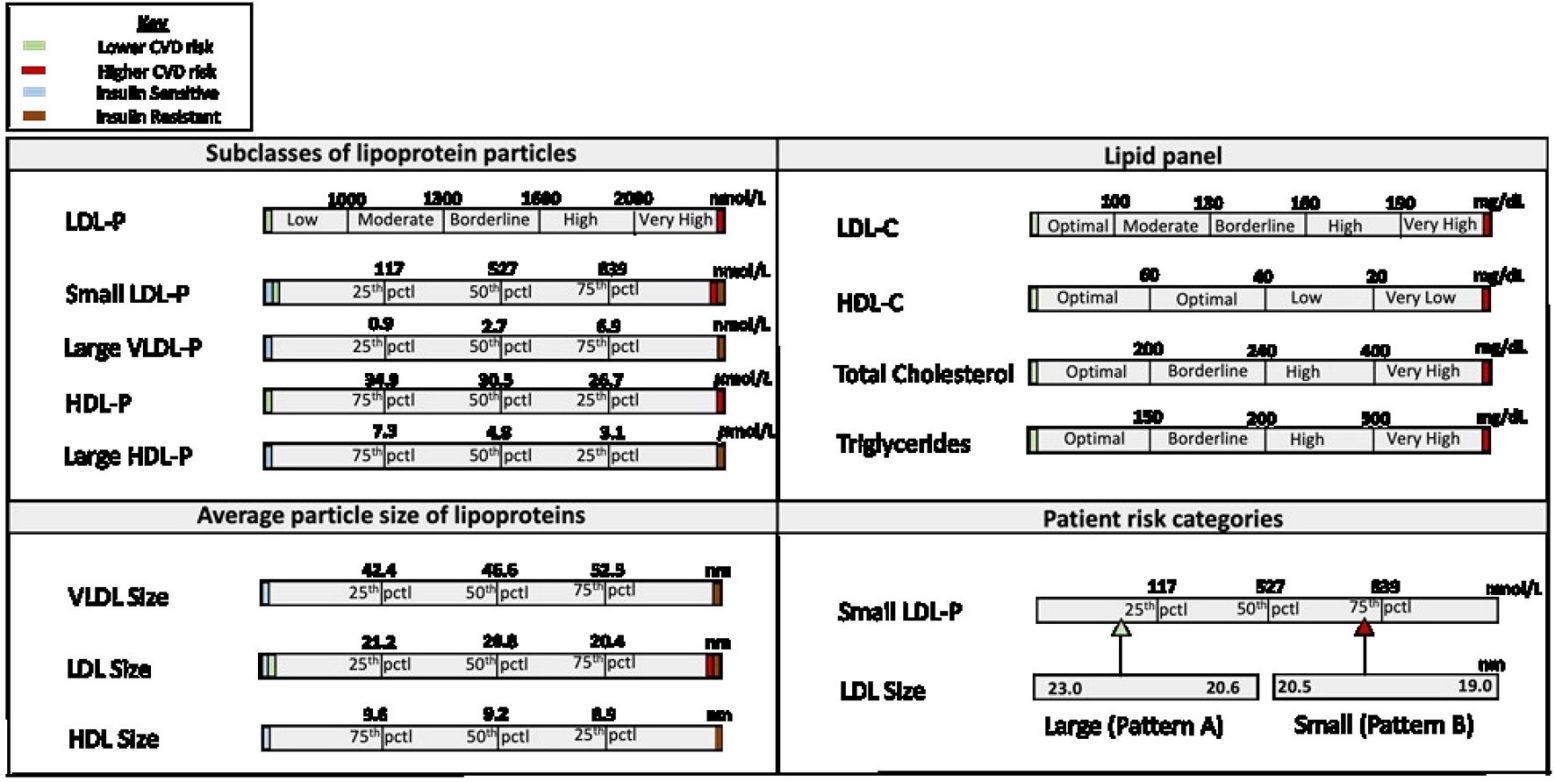
Components of the NMR LipoProfile Test Report. pctl, percentile; CVD, cardiovascular disease; LDL, low density lipoprotein; HDL, high density lipoprotein; VLDL, very low-density lipoprotein; LDL-P, low density lipoprotein particle number; HDL-P, high density lipoprotein particle number; VLDL-P, very low-density lipoprotein particle number; LDL-C, low density lipoprotein cholesterol level; HDL-C, high density lipoprotein cholesterol level.

The NMR LipoProfile test is relatively new and can produce complete lipoprotein profiles uniquely and efficiently through the use of spectroscopy. The technique was decentralized in 2007 and laboratories all over the world could now obtain a fully automated NMR clinical analyzer. Using the analyzer, automated measurement of the patients' serum/plasma proton NMR spectrum is performed, and the resulting digitized spectrum is stored in the computer's memory. Following this, the analysis software extracts the amplitudes of the individual subclass NMR signals and converts them to suitable units of concentration. This data is presented in a spreadsheet or report format depending on the use (23). The process is accurate because the NMR signal emitted by each class of lipoproteins, VLDL, LDL, and HDL, has a unique spectral line shape which makes it possible to differentiate one from the other. Furthermore, accurate quantification of particle number occurs because the amplitudes of the individual subclass NMR signals are directly proportional to the number of particles emitting the signal. Identification of different sizes of the lipid particles is due to a magnetic property specific to lipoproteins which enables the lipids in smaller particles to emit signals that are different in shape and lower in frequency from signals emitted by lipids in larger particles (24). This automated technique can simultaneously measure up to 15 subclasses of lipoproteins. The data generated includes information on 1: subclasses of LDL, HDL, and VLDL; 2: LDL-Cholesterol, HDL cholesterol, total cholesterol, and triglycerides, similar to the lipid panel; 3: average particle sizes of LDL, HDL, and VLDL; and 4: patient risk categories based on their LDL subclass phenotype. Patient risk categories include pattern A (predominantly large LDL, signifying lower CHD risk), pattern B (predominantly small LDL, signifying higher CHD risk), or pattern AB (intermediate category) (Figure 1).

### Advantages of Using the NMR LipoProfile

Using the NMR LipoProfile in clinical settings can be beneficial, particularly in both the assessment and management of an individual's cardiovascular risk. A typical NMR profile report gives a complete picture of the lipoprotein profile including information that is contained in the traditional lipid panel assays as well as information on lipoproteins that are more accurate in assessing cardiovascular risk status. It more accurately categorizes high-risk and low-risk patients when compared to the standard lipid panel tests. This presents a pathway to providing individualized care for patients and reducing the disparities in treatment outcomes, especially in individuals with LDL-C and LDL-P discordance. Therefore, serving as an important tool to aid clinicians in the decision-making process to ensure optimal patient management.

In addition, NMR LipoProfile testing has other clinical applications in assessing the risk of insulin resistance and systemic inflammation. NMR LipoProfile testing can provide information on the Lipoproteins Insulin Resistance index, thus providing valuable information on insulin resistance (25). Identifying patients at risk of developing Insulin resistance and subsequently type 2 diabetes mellitus, a coronary artery disease risk equivalent, will allow for close monitoring and initiation of preventative therapy (25, 26). Furthermore, NMR LipoProfile can incorporate the GlycA test in assessing patients at risk of cardiovascular disease (27). GlycA is an inflammatory biomarker that measures the activities of N-glycosylation of various plasma acute-phase proteins, serving as a marker of systemic inflammation (27, 28).

### Disadvantages of the NMR LipoProfile

There are a few cons to the NMR LipoProfile test. These include associated expenses and long result wait time. On average, the cost of each NMR LipoProfile test ranges from $100 to $450, of which there is often full or at least partial coverage by Medicare and private insurance providers (29, 30). Traditional lipid profile tests are much cheaper, costing between 27 and 36 US dollars (31). Furthermore, the timeline of results for each test varies with the NMR taking more time. The expected turnaround time for lipid panel tests is within 1 day while the turnaround time for NMR LipoProfile tests is between 1 and 3 days (32, 33). Despite these disadvantages, the clinical value of the NMR LipoProfile is significant enough to warrant consideration for use in cardiovascular clinical settings.

## Conclusion

It is apparent that LDL-P are key predictors of cardiovascular risk. In most cases, the higher the number of LDL-P, the higher the risk of cardiovascular disease. This increased risk is also present even when LDL-C level is low. As such, the measurement of LDL-P number is beneficial in all individuals, especially in subsets of people with increased risk but unidentifiable by measuring LDL-C levels. Despite the predictive value of the LDL-P number, treatment goals and clinical decision-making in the management of cardiovascular risk are currently focused on achieving target LDL-C levels (6, 10). NMR LipoProfile, in addition to measuring lipoprotein cholesterol levels, also measures LDL particle number, identifies subsets of individuals who need monitoring, and measures other parameters like insulin resistance markers of systemic inflammation that help predict cardiovascular risk.

With the availability of numerous pieces of evidence supporting the usefulness of the NMR LipoProfile test, it is surprising that it is not widely applied in clinical cardiovascular care. Current guidelines for the management of lipid disorders focus on different parameters related to assays of LDL-C levels or another lipoprotein cholesterol (34). It may be worthwhile to consider expanding guidelines to include NMR assessments of lipoprotein particle number, given how specific it is to the individual patient—especially in instances where there is variability in LDL-C and LDL-P number. NMR LipoProfile also has implications for research. Medications that are used in the management of cardiovascular risk attributable to lipoproteins include moderate- to high-intensity statins, ezetimibe, bile acid sequestrants, niacin, and combination modalities of these drug classes (19, 35). These drugs exhibit variation in their lipid-lowering effect with statins resulting in the highest percent reduction of both LDL-P and LDL-C (19, 35) (see Table 1). Because of the effectiveness of statins, they have become the first-line therapy in the management of lipid disorders. However, the use of statins leads to smaller percent reductions in LDL-P number than in LDL-C (15, 36, 37). It is also important to note that lifestyle modification is associated with improvement in advanced lipoproteins including a reduction in LDL-P number, a reduction in small HDL particle (HDL-P) number, and an increase in the concentration of large HDL-P numbers (38). Future studies that thoroughly examine the impact of different medications on the LipoProfile particle number in comparison with LDL-C will help identify response to therapy and can also result in changes to guidelines for the management of lipid disorders. Additionally, these studies should control for the effect of exercise and other lifestyle modifications in their study designs. Advanced lipoprotein measurements can provide additional insights into selected patients, which can be quite helpful and lead to changes in treatment. Howeverr, more clinical trial data is needed to demonstrate the superiority of utilizing advanced lipoprotein testing on clinical outcomes.

**Table 1:** Effect of lipid lowering therapy on LDL particle number and LDL cholesterol.

**Agent**	**Reduction in LDL-P (%)**	**Reduction in LDL-C (%)**
Moderate- to high-dose statins (simvastatin, pitavastatin, atorvastatin, rosuvastatin)	35–55	27–60
Ezetimibe	15–25	15–20
Bile acid sequestrants	15–30	10–30
Statins + ezetimibe or bile acid sequestrants	50–70	Up to 70%
Statins + niacin	50–70	
Statins + ezetimibe or bile acid sequestrants + niacin	>60	

*Adapted from Cromwell and Triffon (19) and Feingold and Grunfeld (35)*.

Having laid down the facts that the number of the LDL-P is more specific to cardiovascular risk, it is important to thoroughly examine the role of NMR LipoProtein in both inpatient and outpatient settings, and more so of medication therapy in addressing LDL-P. The benefits of using NMR LipoProfile in cardiovascular care far outweigh the disadvantages. Although it is more expensive and takes longer to result, the more widely used the test becomes to practitioners, the likelier that technological advancements and competition between providers will lead to decreased cost and quicker turn-around of results. Nevertheless, using the LDL-P number in the future, as a guide to lipid-lowering therapy, may be cost-effective or even cost-saving for high-risk patients (39).

## Author Contributions

VS and EE conceptualized and designed the study, wrote the original draft, and reviewed and edited the manuscript. VS also supervised the manuscript preparation. EI, IN, NM, and JS reviewed and edited the manuscript. All authors approved the final manuscript as submitted and agree to be accountable for all aspects of the work.

## Conflict of Interest

The authors declare that the research was conducted in the absence of any commercial or financial relationships that could be construed as a potential conflict of interest.

## Publisher's Note

All claims expressed in this article are solely those of the authors and do not necessarily represent those of their affiliated organizations, or those of the publisher, the editors and the reviewers. Any product that may be evaluated in this article, or claim that may be made by its manufacturer, is not guaranteed or endorsed by the publisher.
